# Efficacy of a multidimensional versus usual care physiotherapy on pain and electroencephalography (EEG) spectrum in chronic nonspecific low back pain: study protocol for a randomized controlled trial

**DOI:** 10.1186/s13063-021-05580-3

**Published:** 2021-10-07

**Authors:** Sanaz Bemani, Shohreh Noorizadeh Dehkordi, Javad Sarrafzadeh, Saeed Talebian, Reza Salehi, Jamileh Zarei

**Affiliations:** 1 Department of Physiotherapy, School of Rehabilitation Sciences, Iran University of Medical Sciences, Madadkaran St, Shahnazari St, Madar Sq. Mirdamad Blv, Tehran, Iran; 2 Department of Physiotherapy, School of Rehabilitation Sciences, Tehran University of Medical Sciences, Tehran, Iran; 3 Rehabilitation Research Center, Department of Rehabilitation Management, School of Rehabilitation Sciences, Iran University of Medical Sciences, Tehran, Iran; 4 Department of Health Psychology, School of Behavioral Sciences and Mental Health, Iran University of Medical Sciences, Tehran, Iran

**Keywords:** Chronic low back pain, Biopsychosocial, disability, Fear of movement, Electroencephalography

## Abstract

**Background:**

Non-specific chronic low back pain (NSCLBP) is a major public health and global socioeconomic burden associated with a complex interplay of biopsychosocial factors. Despite scientific signs of progress, treatment of NSCLBP often tends to stick to a biomechanical model, without targeting psychological and social factors. To enhance the clinical efficacy of usual physiotherapy for NSCLBP, the development of clinical strategies is to be pursued. This study aims to assess the effectiveness of multidimensional physiotherapy based on a biopsychosocial approach compared to usual care physiotherapy, on clinical findings and electroencephalography spectrum in non-specific chronic low back pain.

**Methods:**

This study is a triple-blind, two-arm (1:1) randomized controlled trial with a 4 months follow-up. Seventy NSCLBP patients will be randomly allocated to either the experimental (multidimensional physiotherapy) or the active control group (usual physiotherapy); each group will receive 6 weeks of physiotherapy. The main outcome is pain and secondary outcomes are brain function, quality of life, disability, lumbar flexion range of motion, and psychosocial correlates. Assessment will be performed at baseline, post-treatment, and at 1 and 4 months follow-up.

**Discussion:**

Findings may provide evidence on the effectiveness of multidimensional physiotherapy on clinical findings and brain characteristics and might provide evidence towards showing the role of brain and biopsychosocial factors on chronic pain.

**Trial registration:**

ClinicalTrials.gov NCT04270422, Registered on 17 February 2020, IRCT Identifier: IRCT20140810018754N11

## Administrative information

**Table:** 

Title {1}	Efficacy of a Multidimensional versus usual physiotherapy on pain and Electroencephalography (EEG) Spectrum in Nonspecific Chronic Low Back Pain: Study protocol for a Randomized Controlled Trial
Trial registration {2a and 2b}.	ClinicalTrials.gov Identifier: NCT04270422, Registered 17 February 2020, https://clinicaltrials.gov/ct2/show/NCT04270422?term = NCT04270422&draw = 2&rank = 1 IRCT Identifier: IRCT20140810018754N11
Protocol version {3}	Version 1.0, Oct 2020
Funding {4}	This research received no specific grant from any funding agency in the public, commercial or not-for-profit sectors.
Author details {5a}	‘SB’ PhD Candidate, Physiotherapy, Department of Physiotherapy, School of Rehabilitation Sciences, Iran University of Medical Sciences, Tehran, Iran. ‘ShN’ PhD Physiotherapy, Associate Professor, Department of Physiotherapy, School of Rehabilitation Sciences, Iran University of Medical Sciences, Tehran, Iran. ‘JS’ PhD Physiotherapy, Professor, Department of Physiotherapy, School of Rehabilitation Sciences, Iran University of Medical Sciences, Tehran, Iran. ’ST’ PhD Physiotherapy, Professor, Department of Physiotherapy, School of Rehabilitation Sciences, Tehran University of Medical Sciences, Tehran, Iran. ‘RS’ PhD Physiotherapy, Associate Professor, Rehabilitation Research Center, Department of Rehabilitation Management, School of Rehabilitation Sciences, Iran University of Medical Sciences, Tehran, Iran. ’JZ’ PhD Health Psychology, Assistant Professor, Department of Health Psychology, School of Behavioral Sciences and Mental Health, Iran University of Medical Sciences, Tehran, Iran. All Authors read and approved the manuscript.
Name and contact information for the trial sponsor {5b}	Not applicable
Role of sponsor {5c}	Not applicable

## Introduction

### Background and rationale {6a}

Low back pain (LBP) is the most frequent self-reported, costly, and debilitating type of musculoskeletal pain that imposes significant health and economic problems [1, 2]. The prevalence of LBP estimates 33% for point prevalence, 65% for 1 year prevalence, and 84% for lifetime prevalence [3, 4]. Eighty to 90% of LBP is self-limiting, while almost 20% of the cases become persistent and disabling and negatively affect many aspects of daily life [3, 5, 6]. Evidence shows that only 8–15% of patients with LBP have an identified pathoanatomical diagnosis (specific LBP), resulting in the majority being diagnosed as having non-specific LBP [7]. Among this non-specific population, a small but considerable group becomes chronic and disabled, which is labeled non-specific chronic low back pain (NSCLBP) [1, 8]. The effective management of NSCLBP is a major concern for people, the economy, and society as a whole [1, 7, 9]. NSCLBP is no longer considered a purely structural, anatomical, or biomechanical disorder of the lumbar spine. Instead, there is strong evidence that NSCLBP is associated with a complex interaction of factors involve structural or biomechanical, cognitive (e.g., unhelpful beliefs, catastrophizing, maladaptive coping strategies, low self-efficacy), psychological (e.g., fear, anxiety, depression), lifestyle (e.g., immobility, sleep problems), and social (e.g., work and family issues) factors [1, 8, 10–13]. Consequently, NSCLBP treatment guidelines generally acknowledge a shift from biomedical models to the biopsychosocial model. In this approach, the cognitive, psychological, and social dimensions of pain are adopted in addition to the physical and pathoanatomical dimensions [1, 6, 14].

The perception of pain is the result of a complex, dynamic system. The relationship between nociceptive information and pain is profoundly impressed by affective and cognitive factors. A specific region of the cortex that is responsible for pain processing has not yet been found; however, a complex network of brain regions known as a pain matrix has been identified. According to the neuromatrix theory of pain, chronic pain is considered a multidimensional experience and needs new forms of multidimensional treatment. The area of the brain involved in pain experience is a large and widespread network of neurons that consists of loops between the thalamus and cortex, as well as between the cortex and limbic system. These loops are not just for pain perception but also are involved in other sensory, motor, and cognitive functions [15, 16]. Functional MRI studies have shown that spontaneous back pain in CLBP patients does not activate the areas of the pain matrix typically seen during noxious stimulation, which supports the notion that chronic pain may not be caused by the nociceptive process [17].

Although growing knowledge exists on the peripheral and spinal neuronal mechanism of pain chronification processes, little is known about the accompanying neural mechanisms of chronic pain [18]. Recent studies have identified a relationship between chronic neurogenic pain and the presence of specific electroencephalography (EEG) pattern called thalamocortical dysrhythmia (TCD). TCD is characterized by a common oscillatory pattern in which resting-state alpha activity is replaced by cross-frequency coupling of low- and high-frequency oscillations. Despite many advances in the treatment of chronic pain, major challenges remain, and the main cause is the lack of precise diagnosis of the underlying cause of chronic pain. The somatosensory cortex and thalamocortical loop appear to be the best candidate for understanding chronic pain because these regions are most active in processing sensory information and have a strong role in processing pain [18–20].

Various non-surgical treatments have been suggested for NSCLBP. These include non-steroidal anti-inflammatory drugs, patient education, hydrotherapy, manual therapy, physiotherapy, acupuncture, and cognitive-behavioral therapy [1, 9]. Physiotherapy is one of the common interventions for the management of LBP [21–26]. General exercises and patient education have the most evidence and use as usual physiotherapy [9, 27–29]. Although routine physiotherapy helps patients recover, about one third of patients run a recurrence of LBP within a year. We hypothesize that usual LBP physiotherapy relies solely on the biomechanical dimension of health and ignores the psychosocial aspects [3, 22, 30, 31].

In the present study, the effect of a multidimensional treatment based on the biopsychosocial approach will be compared with a usual care physiotherapy treatment on pain and function, brain function, and pain psychosocial correlates in chronic nonspecific low back pain.

### Objectives {7}

The overall objective of this study is to demonstrate the impact of multidimensional treatment in the management of CNSLBP. The specific objectives are:

1. Primary objective: Effect of a multidimensional treatment on pain compared to usual care physiotherapy
2. Secondary objectives:

- Effect of a multidimensional treatment on brain function compared to usual care physiotherapy
- Effect of a multidimensional treatment on lumbar flexion ROM compared to usual care physiotherapy
- Effect of a multidimensional treatment on quality of life compared to usual care physiotherapy
- Effect of a multidimensional treatment on psychological correlates of pain compared to usual care physiotherapy

### Trial design {8}

**Fig. 1:**
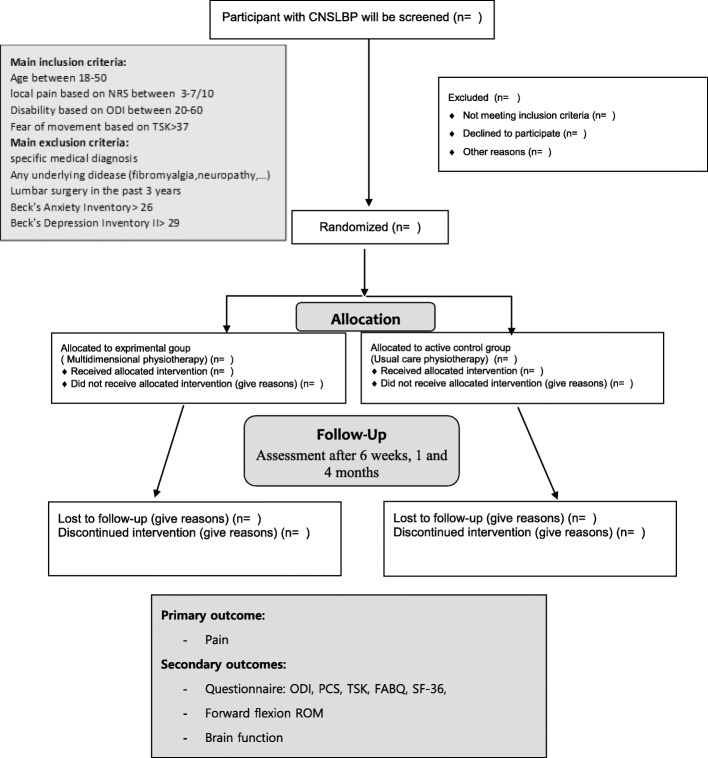
Flow chart of research design (CNSLBP, chronic non-specific low back pain; NRS, Numerical Rating Scale; PCS, Pain Catastrophizing Scale; SF-36, Medical outcomes 36 Health Service Survey; TSK, Tampa Scale for Kinesiophobia; ODI, Oswestry Disability Index; FABQ, Fear Avoidance Beliefs Questionnaire; ROM, range of motion)

The present study is a 4-month, triple-blind, randomized, controlled, parallel trial that will be carried out between December 2020 and November 2022. Patients with NSCLBP will be enrolled in a 6-week physiotherapy program organized in IRAN (Iran University of Medical Science). More specifically multidimensional physiotherapy will be compared to usual physiotherapy. The outcome will be assessed at baseline, after 6 weeks of treatment, and at 1 and 4 months follow-up. The participant flowchart through the study is shown in Fig. 1.

## Methods: participants, interventions, and outcomes

### Study setting {9}

The study population will include approximately 70 CNSLBP patients. Participants will be recruited by the participating research groups from the Iran University of Medical Science’s hospitals and via adverts. This study will be conducted in the physiotherapy clinic in the School of Rehabilitation Sciences at the Iran University of Medical Sciences, Tehran, Iran.

### Eligibility criteria {10}

Patients will be eligible for this study if they have the following: age between 18 and 50, permanent or intermittent local pain between L1 to gluteal fold without any radicular pain for 3 months or more and between 3 and 7/10 based on the Numeric Rating Scale (NRS), disability based on the Oswestry Disability Index (ODI) between 20 and 60, fear of movement based on the Tampa scale for Kinesiophobia > 37, elementary level of education, and Native Persian speaking. The most important exclusion criteria are as follows: any evidence of specific medical diagnosis include spondylolisthesis, fracture, tumor, or inflammation disease; rheumatoid disease, fibromyalgia, neuropathy, and progressive neurological disease; history of headache, dizziness, nausea, epilepsy, migraines, and mental disorders; history of lumbar surgery in the past 3 years; the Beck Anxiety Inventory > 26; the Beck Depression Inventory II > 29; pregnancy; and having other therapies during the present research. The drop-out criteria are as follows: the presence of any condition that interferes with the intervention or assessment, participate in any other treatment during the interventions, inclusion or exclusion criteria are violated, and a patient requests to be removed from the trial.

### Who will take informed consent? {26a}

At an in-person meeting, the investigator who performs the assessments will explain the study details and clarify any questions. Then, the participant will be asked to sign the consent form. It consists of the details, such as the title of the study, names of investigators, registered information, research background, how the study was to be conducted, what the participants should do in the study, treatment plans, and obligations.

### Additional consent provisions for collection and use of participant data and biological specimens {26b}

Not applicable.

## Interventions

### Explanation for the choice of comparators {6b}

After obtaining written informed consent, baseline measurements will be performed. Participants will then be randomized to either active control or experimental group (1:1 ratio) using a stratified block allocation with stratification factors being gender (male or female) and with a block size of four. Randomization will be done at the Iran University Medical Sciences by an independent investigator using WinPepi (version 11.65). The randomization schedule will be known only to 1 investigator who is not involved in recruiting participants. The randomization will be concealed from patients and the other investigators involved in patient assessments, treatments, and analyses.

### Intervention description {11a}

All interventions are performed by two expert and trained physiotherapists with 6 years of work experience in the treatment of LBP patients. The intervention lasts six weeks and each group will receive 12 sessions of physiotherapy.

#### Experimental group

Patients in the experimental group will receive multidimensional physiotherapy include psychoeducation based on cognitive behavioral therapy (CBT), lifestyle education, graded exposure, postural correction exercise, and electrotherapy. Twelve 30-min psychoeducation sessions consist of the following sessions:

1. Psychoeducation on pain based on the book “Explain Pain” [32] and set the goal of treatment (3 sessions);
2. Anxiety management: The goal of this session is to identify signs and symptoms of anxiety and to learn coping skills to improve the individual’s functioning in the area of anxiety.
3. Interpersonal conflict management: The goal of this session is to have individuals learn how to manage conflict more effectively and increase effective skill use in relationships.
4. Problem-solving training: The goal of this session is to promote problem-solving strategies in complex situations and with others.
5. Coping strategy training: The goal of this session is to deliver insight and information about coping strategies for chronic pain and to learn coping skills for improving patient functioning in the areas of common defense mechanisms.
6. Pain flare-ups management: This session is run to identify triggering events and typical reactions and promote coping skills with the fear and avoidance conditioning.
7. Medication abuse management: This session is held to learn coping skills to improve the individual’s functioning in accepting reality and learn coping skills to improve the individual’s functioning in chemical abuse.
8. Enhancing one’s ability to cope with labeling and stigma: The goal of this session is to give insight and understanding into the effects of stigma and learn coping skills to improve the individual’s ability to cope effectively with stigma.
9. Empowering one to create a daily sleep routine: The goal of this session is to learn coping skills to improve the individual’s functioning in the areas of building and maintaining healthy sleep patterns.
10. Training relaxation techniques [33].

Patients will also receive recommendations for lifestyle modifications, such as encouraging active living and a gradual increase in activity levels, improving the work environment, and training on how to perform daily and work activities correctly. Other treatments include postural correction and training of the necessary exercises, as well as the gradual initiation of movements and activities that the patient is afraid of, and by asking the patient to perform hierarchical activities that he or she fears. The first step is to identify the movements that the patient is afraid of. The exercise begins with a movement that the patient has the least fear of, and that movement begins with the simplest state and the lowest level with the patient least feared, and gradually becomes more difficult [34, 35]. All of these are taught to the patient in each practice session. The patient will also be asked to repeat the practice of each session at home once a day. Electrotherapy, including 20 min of transcutaneous electrical nerve stimulation (TENS) at 100 Hz in the lumbar region where the patient reports the most pain, will also be done. The intensity of TENS is regulated by the patient. Surface heat will be placed in the lumbar area by Hot Pack at the same time as TENS. Ultrasound with a frequency of 1 MHz and intensity of 1.2 W/cm^2^ will be performed using a 5-cm square applicator on the paravertebral muscle where the patient reports the most pain, for 5 min. The treatment in this group is twelve sessions and will be performed twice a week.

#### Active control group

For patients in the active control group, treatment will include education, trunk general exercises, and electrotherapy. Education in this group includes an explanation of the basic biomechanical anatomy of the spine, common causes of spinal pain, pain processing, ergonomic advice on how to perform daily activities, and postures (such as sitting, standing, moving objects, etc.), and training for postural correction exercises. General trunk exercise will focus on abdominal and paraspinal muscles to improve blood flow, mobility, strength, and endurance in each session, and the patient will be asked to perform exercises in each session [36]. The intensity and type of exercise will progress toward functional training. The patient is also asked to do exercise at home once a day. Electrotherapy, including 20 min of skin electrical nerve stimulation (TENS) at 100 Hz in the lumbar region where the patient reports the most pain, will also be done. The intensity of TENS is regulated by the patient. Surface heat will be placed in the lumbar area by Hot Pack at the same time as TENS. Ultrasound with a frequency of 1 MHz and intensity of 1.2 W/cm^2^ will be performed using a 5-cm square applicator on the paravertebral muscle where the patient reports the most pain, for 5 min. The treatment in this group is twelve sessions and will be performed twice a week.

### Criteria for discontinuing or modifying allocated interventions {11b}

Not applicable

### Strategies to improve adherence to interventions {11c}

Not applicable

### Relevant concomitant care permitted or prohibited during the trial {11d}

Relevant concomitant care and interventions are prohibited during the trial.

### Provisions for post-trial care {30}

No physical or psychological damage has been predicted in this study; however, any possible problem, either physical or mental, which arises during the research, due to the treatment will be reported to the Iran University of Medical Sciences ethics committee. The study team will give priority to unpaid examination and treatment according to the regulations of the Ethics Committee.

## Outcomes {12}

The pain will be the primary outcome. The secondary outcomes will include quality of life, fear avoidance beliefs, kinesiophobia, pain catastrophizing, disability, forward flexion ROM, and absolute and relative power of the frequency spectrum of EEG.

### Primary outcome

The pain will be assessed through a self-reported questionnaire. This questionnaire will be used for pain assessment at baseline, post-treatment, and at 1 and 4 months follow-up:

- A Numerical Rating Scale (NRS) for pain ranging from 0 “no pain at all” to 10 “worst imaginable pain” (“How would you rate your low back pain, on average, over the last 3 days?”) [37].

### Secondary outcomes

#### Psychosocial correlates

Patients will complete some questionnaires at baseline, after 12 treatment sessions, and at 1- and 4-month follow-ups. The following standardized and reliable questionnaires (Persian version) will be used to measure psychological factors that may interfere with pain:

- Self-reported pain catastrophizing based on the Pain Catastrophizing Scale. The Pain Catastrophizing Scale (PCS) will be included to assess catastrophic thinking about pain. It consists of 13 items describing different thoughts and feelings that individuals may have when experiencing pain. Items are scored on a 5-point scale, 0 “not at all” to 4 “all the time.” A general score and scores on 3 subscales (i.e., helplessness, magnification, and rumination) will be obtained; higher scores indicate more severe catastrophic thoughts about pain [38, 39].
- Self-reported kinesiophobia based on the Tampa scale of Kinesiophobia. The Tampa Scale for Kinesiophobia (TSK) is a 17-item questionnaire that will be used to measure the fear of(re) injury due to movement. Scores range from 17 to 68, with scores ≤ 37 suggesting low fear of movement and scores > 37 indicating high fear of movement. The TSK-Persian version that will be used in this study is shown reliable and valid [40, 41].
- Self-reported fear avoidance beliefs based on the Fear Avoidance Beliefs questionnaire. The Fear Avoidance Beliefs questionnaire (FABQ) is a 16-item self-reporting questionnaire evaluating patient’s attitudes and beliefs toward the effect of physical activity and work on their pain. The FABQ is divided into two subscales which are designed to assess beliefs about work and physical activity. The agreement of patients with each item will be scored by a 7-point Likert scale, ranging from 0 (completely disagree) to 6 (completely agree). Higher FABQ scores indicate higher fear-avoidance beliefs [42, 43].

#### Quality of Life

- The 36 Health Status Survey (SF-36) will be used to assess functional status and well-being or quality of life. The SF-36 contains 8 dimensions (physical functioning, social functioning, physical role, emotional role, mental health, vitality, bodily pain, and general health perceptions). The overall value ranges from 0 to 100, with improvement as scores increase. The psychometric properties of the SF-36 are well-characterized in a wide variety of patient populations [44–46].

#### Disability

- Self-reported disability based on the Oswestry Disability Index Questionnaire. The Oswestry Disability Index is currently considered for measuring the degree of disability in patients with low back pain. The questionnaire contains ten topics concerning the intensity of pain, lifting, ability to care for oneself, ability to walk, ability to sit, sexual function, ability to stand, social life, sleep quality, and ability to travel. Each question is scored on a scale of 0–5 with the first statement being zero and indicating the least amount of disability and the last statement is scored 5 indicating the most severe disability. The scores for all questions answered are summed and a higher score indicates greater disability [47, 48].

#### Forward flexion range of motion (ROM)

Forward flexion ROM will be measured by the third finger to ground distance by a meter.

#### Brain function

In this study, EEG will be recorded (bandwidth is 0.2–70 Hz, the impedance is less than 20 kHz, and the sampling rate is 512 Hz) with 64 channel amplifiers (EB Neuro, Italy) according to the international 10/20 system, from 19 Ag/AgCl surface electrodes and a diameter of 8 mm electrode sites distributed throughout the whole head of the participants. The 15 electrodes will be placed in Fp1, Fp2, F7, F3, Fz, F4, F8, T3, C3, Cz, C4, T4, T5, P3, Pz, P4, T6, O1, and O2. TCD will be performed in the above areas in the frequency range of delta, theta, alpha, beta, and gamma, and the relative and absolute power levels of each will be evaluated. A reference electrode is placed on the mastoid process and a ground electrode is placed on the Fpz region. To record vertical and horizontal electrooculogram, a pair of electrodes above and below the right eye and another pair are placed on either side of each eye. With the electrooculogram and the O1 and O2 electrodes, blinking and eye movement artifacts are controlled. EEG will be recorded under two conditions: while subjects rested with their eyes open for 3 min and while they will forward flexed in 20 s.

## Participant timeline {13}

**Fig. 2:**
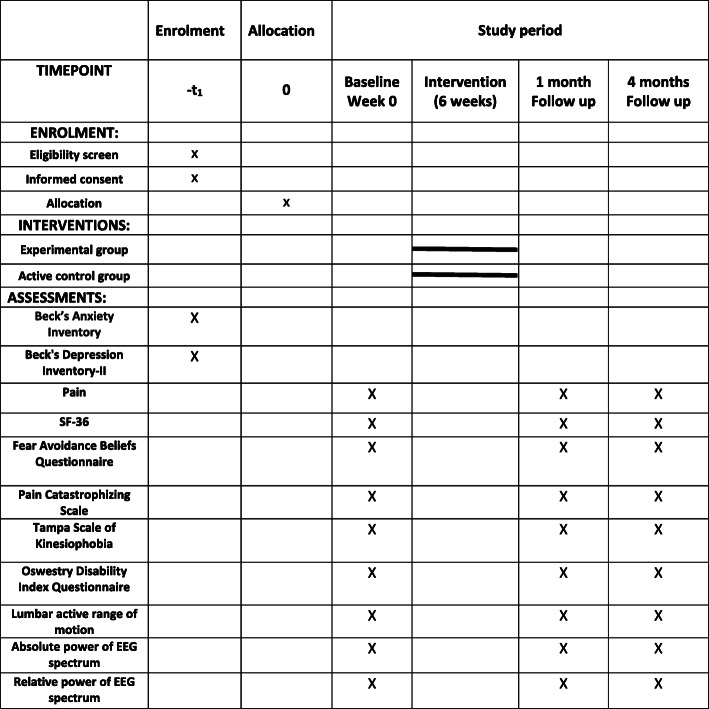
Schedule of enrolment, interventions, and assessments (according to SPIRIT)

This study will be carried out between December 2020 and November 2022. Figure 2 shows the schedule of enrollment, interventions, and assessments (according to the SPIRIT).

### Sample size {14}

Sample size calculations were performed with G*Power 3.1.9.4 (Düsseldorf, Germany) based on the therapy effects on pain and accounting for a 10% loss to follow-up after 4 months. Calculations were based on one-tailed testing with alpha set in 0.025, partial η2 = 0.04, and the desired power of 0.95. The allocation ratio (N2/N1) was defined as 1, resulting in 35 patients in the intervention group and 35 in the control group.

### Recruitment {15}

We will recruit participants through practitioners from Iran University of Medical Science’s hospitals and clinics. Also, we will use advertising posters in each center and online advertisements on the media. Then, the participants will be referred to the outpatient orthopedic rehabilitation center at the School of Rehabilitation Sciences. They will be screened for inclusion and exclusion criteria and make the final decision regarding the eligibility of the patients.

## Assignment of interventions: allocation

### Sequence generation {16a}

After eligibility has been confirmed, patients will be informed about the study comparing two physiotherapeutic treatment options. After obtaining written informed consent, baseline measurements will be performed. Participants then will be randomized to either active control or experimental group (1:1 ratio) using a stratified block allocation with stratification factors being gender (male or female) and with a block size of four. Randomization will be done at the Iran University Medical Sciences by an independent investigator using WinPepi (version 11.65). The randomization schedule will be known only to 1 investigator who is not involved in recruiting participants.

### Concealment mechanism {16b}

The randomization will be concealed from patients, and the other investigators involved in patient assessments, treatments, and analyses.

### Implementation {16c}

Allocation sequence, participant enrollment, and participant assignment to interventions will be performed by an independent investigator who is not involved in assessments, interventions, and any other part of research.

## Assignment of interventions: blinding

### Who will be blinded {17a}

The present study is a triple-blind randomized controlled trial. The patients, investigator, and outcomes assessor will be blind to the treatment groups. To keep patients unaware of any expected treatment group benefit, patients will be informed that the effect of 2 well-established therapies is to be evaluated. An independent and blinded assessor will perform the baseline and follow-up assessments. The researcher who will perform the statistical analyses will not be involved in taking the measurements. The treating physiotherapists will be blinded to the results of the measurements and questionnaires.

### Procedure for unblinding if needed {17b}

Not applicable

## Data collection and management

### Plans for assessment and collection of outcomes {18a}

Not applicable

### Plans to promote participant retention and complete follow-up {18b}

Not applicable

### Data management {19}

Not applicable

### Confidentiality {27}

Not applicable

## Plans for collection, laboratory evaluation, and storage of biological specimens for genetic or molecular analysis in this trial/future use {33}

Not applicable

## Statistical methods

### Statistical methods for primary and secondary outcomes {20a}

Data analysis will be performed using SPSS (version 22) and STATA (version 14.2). Baseline characteristics will be summarized with standard descriptive statistics. Categorical variables will be described with percent and counts. Continuous variables will be described with means and standard deviations. Statistical analysis of the primary and secondary outcomes will be based on the intention-to-treat principle including all randomized patients using an analysis of variance/covariance (ANOVA/ANCOVA). A model including baseline as continuous covariate and group as fixed factors will be used with using Bonferroni post hoc analyses and adjusting for differences in patients’ characteristics at baseline where appropriate will be done. Subgroup analysis of pain catastrophizing variable will be done. For all statistical tests, the significance level will be set at 0.05. Mean difference and standardize Mean difference and their 95% confidence intervals (Cohen’s *D*) will be calculated as are effect sizes.

### Interim analyses {21b}

Not applicable

### Methods for additional analyses (e.g., subgroup analyses) {20b}

Subgroup analysis of pain catastrophizing variable will be done.

### Methods in analysis to handle protocol non-adherence and any statistical methods to handle missing data {20c}

Not applicable

### Plans to give access to the full protocol, participant level-data and statistical code {31c}

Not applicable

## Oversight and monitoring

### Composition of the coordinating center and trial steering committee {5d}

This trial gained approval from the Ethics Committee of the Iran University of Medical Sciences. The ethics number is IR.IUMS.REC.1398.1041.

### Composition of the data monitoring committee, its role and reporting structure {21a}

Given the short period of the intervention and low risk of the trial, DMC will not be formed.

### Adverse event reporting and harms {22}

No physical or psychological damage was predicted in this study; however, any possible problem, either physical or mental, which arises during the research, due to the treatment will be reported to the Iran University of Medical Sciences Ethics committee. The study team will give priority to unpaid examination and treatment according to the regulations of the Ethics Committee.

### Frequency and plans for auditing trial conduct {23}

Independent data monitoring and auditing are not considered because this is a low-risk intervention.

### Plans for communicating important protocol amendments to relevant parties (e.g., trial participants, ethical committees) {25}

If the research plan needs to be modified, it shall provide relevant explanatory materials and application to the Ethical Committee, and the modification can only be made after approval.

### Dissemination plans {31a}

All data or results of the present study will be presented in an article or some articles that will be published after completing the study.

## Discussion

This study aims to compare usual care physiotherapy and multidimensional physiotherapy in CNSLBP patients. The main study question is multidimensional physiotherapy is effective in reducing pain associated with CNSLBP. Further objectives are as follows: to evaluate the effect on brain function, disability, quality of life, lumbar forward flexion ROM, and psychosocial factors related to pain with the inclusion of 70 patients with CNSLBP. The patients, assessors performing the baseline and follow-up evaluations, and the researcher performing statistical analyses are blinded to group allocation. Hence, the study is designed in a way that minimizes potential biases. It is expected that this randomized controlled trial will provide novel data on the effectiveness of a multidimensional when compared to usual care physiotherapy on key patient-centered outcome measures.

In the past studies, most therapeutic approaches to the treatment of chronic low back pain were consistent with biomedical models. In these models, the focus is on structural and biomechanical disorders. Recent studies have shown that pain has sensory, behavioral, and cognitive components, and the association of chronic low back pain with cognitive, behavioral, and social factors has been established. As a result, new biopsychosocial models for the treatment of chronic pain have been introduced, but the implementation of these approaches is not yet common, and the number of studies that have examined the multidimensional treatment of low back pain has been limited. Research has shown that people with chronic low back pain undergo cognitive and emotional changes, but it is unclear what functional changes occur in the central nervous system, especially the brain, and our information is limited.

This study aims to investigate the effect of a multidimensional treatment in comparison with usual physiotherapy on clinical and functional outcomes in patients with nonspecific chronic low back pain. Also, in this study, we evaluated the effects of treatment on the central nervous system and changes in thalamic cortical dysrhythmia pattern in the cerebral band spectra (quantitative evaluation), and we are hopeful that this 4-month prospective trial may contribute towards refining guidelines for good clinical practice and may be used as a basis for health authorities’ recommendations.

## Trial status

This trial was using protocol version 1.0 (February 17, 2020) at the time of this submission. Recruitment started on December 1, 2020, and is expected to be completed on November 30, 2022.

## Data Availability

Data sharing does not apply to this article as no datasets were generated or analyzed during the current study. Individual participant data is available from the corresponding author on reasonable request. The investigators plan to share the results for publication in scientific journals, as soon as study period completion and data are valid and reliable. Study results will also be shared on ClincialTrials.gov.

## Abbreviations

EEG: Electroencephalography
NSCLBP: Non-specific chronic low back pain
NRS: Numerical Rating Scale
PCS: Pain Catastrophizing Scale
SF-36: Medical outcomes 36 Health Service Survey
TSK: Tampa Scale for Kinesiophobia
ODI: Oswestry Disability Index
FABQ: Fear Avoidance Belief Questionnaire
ROM: Range of motion
